# Expression of complement system components during aging and amyloid deposition in APP transgenic mice

**DOI:** 10.1186/1742-2094-6-35

**Published:** 2009-11-17

**Authors:** Julia Reichwald, Simone Danner, Karl-Heinz Wiederhold, Matthias Staufenbiel

**Affiliations:** 1 Novartis Institutes for BioMedical Research, Forum1, Novartis Campus, CH-4056 Basel, Switzerland

## Abstract

**Background:**

A causal role of the complement system in Alzheimer's disease pathogenesis has been postulated based on the identification of different activated components up to the membrane attack complex at amyloid plaques in brain. However, histological studies of amyloid plaque bearing APP transgenic mice provided only evidence for an activation of the early parts of the complement cascade. To better understand the contribution of normal aging and amyloid deposition to the increase in complement activation we performed a detailed characterization of the expression of the major mouse complement components.

**Methods:**

APP23 mice expressing human APP751 with the Swedish double mutation as well as C57BL/6 mice were used at different ages. mRNA was quantified by Realtime PCR and the age- as well as amyloid induced changes determined. The protein levels of complement C1q and C3 were analysed by Western blotting. Histology was done to test for amyloid plaque association and activation of the complement cascade.

**Results:**

High mRNA levels were detected for C1q and some inhibitory complement components. The expression of most activating components starting at C3 was low. Expression of C1q, C3, C4, C5 and factor B mRNA increased with age in control C57BL/6 mice. C1q and C3 mRNA showed a substantial additional elevation during amyloid formation in APP23 mice. This increase was confirmed on the protein level using Western blotting, whereas immunohistology indicated a recruitment of complement to amyloid plaques up to the C3 convertase.

**Conclusion:**

Early but not late components of the mouse complement system show an age-dependent increase in expression. The response to amyloid deposition is comparatively smaller. The low expression of C3 and C5 and failure to upregulate C5 and downstream components differs from human AD brain and likely contributes to the lack of full complement activation in APP transgenic mice.

## Background

The complement system is a major effector of the humoral immune system playing an important role in both innate and acquired immunogenicity [1]. It consists of various proteins acting in different but merging cascades, the main ones being the classical and the alternative pathway. Most complement protein is synthesized in the liver, but in brain microglia, astrocytes and also neurons have been implied in complement synthesis as well [2-4].

A causal role of the complement system in Alzheimer's disease pathogenesis has been postulated previously (for a summary see [5-7]). Histological studies of AD brain detected complement components in association with parenchymal amyloid plaques but also neurofibrillary tangles [8]. This includes key components for activation up to the ultimately formed membrane attack complex [9-15]. Moreover, an increase in brain complement RNA expression was described [16]. In biochemical assays, fibrillar or oligomeric Aβ and aggregated tau have been demonstrated to activate the classical and alternative complement pathways suggesting an induction of the system by amyloid deposits in vivo [8,17-20]. Histological studies of amyloid plaque bearing APP transgenic mice detected early complement components, whereas downstream components forming the membrane attack complex were missing [21]. The first component of the classical pathway, C1q, could be detected in association with plaques in an age-dependent manner in APP and APP/PS1 transgenic mice and was upregulated in plaque-associated microglia [22]. While it has been argued that human Aβ may be a poor activator of mouse complement, humanization of the C1q A chain did not increase activation] [23,24].

When crossing mice deficient for C1q and APP transgenic animals, Fonseca and co-workers [25] observed a reduction in amyloid associated glial and neuritic pathology, whereas amyloid deposition seemed unaffected. In contrast, knock out of C3, at which the classical and alternative pathways merge, or overexpression of its inhibitor Crry increased the amyloid load and neurodegeneration but reduced phagocytic microglia [26,27]. A reduction in neuritic pathology was also found in APP transgenic mice lacking clusterin (apolipoprotein J), a regulator of the complement system [28,29], possibly due to a shift towards non-fibrillar Aβ deposits. More recently, treatment of APP transgenic mice with a C5a receptor antagonist was shown to reduce fibrillar amyloid and gliosis together with improved synaptophysin staining and behavioural performance [30]. While these functional studies suggest a role of complement in amyloid bearing mouse brain, the partially contrasting effects of C1q and C3 blockade are remarkable. This may indicate activities which are independent of full complement activation [7], in particular since evidence for an activation of downstream components is missing. On the other hand, the incomplete complement activation in APP transgenic mice raises doubts in the relevance of the neuroprotective effects for human AD brain. Although the mentioned data overall suggest a deleterious role, the significance of complement activation in AD brain remains unclear at present.

To better understand the age- and amyloid-related changes in the complement profile of mouse brain we have analyzed the expression of the major components during aging of C57BL/6 mice as well as during amyloid deposition in the APP transgenic mouse line APP23.

## Methods

APP23 mice overexpressing human APP751 with the Swedish double mutation [31] as well as C57BL/6 (non-transgenic littermates) and BUB/BnJ [32] mice were analyzed at the age of 3-30 months. mRNA was quantified by Realtime PCR [33], proteins were analyzed by Western blotting and histology. For comparison of expression levels human samples were analyzed likewise. The human samples were obtained from the Institute of Pathology, University of Basel, Switzerland as approved by the ethics commission of Basel.

### Animals

Female APP23 mice (B6, D2-Tg(Thy1App)23/1Sdz) and non transgenic littermates at 3, 6, 9, 12, 15, 18, 24 and 30 months were obtained from Novartis Pharma AG, Special Breeding, Basel, St. Johann, Switzerland. BUB/BnJ mice at 2 months were obtained from Jackson Laboratories, Bar Harbor, USA. Mice were kept under standard conditions. The study was done under animal permission No. 1795 as issued by the Kantonales Veterinäramt Basel-Stadt.

### Forebrain, brain, liver and serum preparation

For forebrain and serum preparation the animals were anaesthetized with 3.5% (volume) isoflurane (FORENE; Abott Laboratories, #B506) in a narcotic chamber and decapitated immediately. The blood was collected in microtainers (BD Bioscience, #365951) and used to prepare serum samples. Blood samples were kept at RT for 30 min, centrifuged (4°C, 6800 g, 15 min) to obtain the sera and stored at -80°C until they were used for Western blots. The brains were removed and separated into left and right hemisphere. Forebrains were separated from cerebellum and brain stem and immediately frozen on a metal plate on dry ice. From some animals the liver was removed and frozen in the same way. Samples were stored at -80°C until use for RNA isolation or Western blots.

The frontal cortex region (F2) was obtained at autopsy from 3 individuals without a history of neurological disease and AD pathology (1 female and 2 males; age 61 - 78 years; post mortem delay 3.5 - 12 hours). The tissues were frozen at -80°C until use.

### PBS perfusion of mice

Pentobarbital anaesthetized (50 mg/ml Nembutal; Abbott Laboratories, #6900) mice were perfused transcardially with 0.01 M PBS pH 7.4 (Sigma, #P-4417) for about 8 minutes at room temperature. Brains were removed from skull, separated into left and right hemisphere, separated from cerebellum and brain stem, immediately frozen on a metal plate on dry ice and kept at -80°C until use.

### Total RNA extraction and cDNA synthesis

For total RNA extraction from frozen forebrains the RNeasy Lipid Tissue Mini Kit from QIAGEN (#74804) was used according to the manufacturer's instructions. The tissue was homogenized in the Retsch Mill MM 300 (QIAGEN, #85120) for 2 min at 20 Hz followed by 1 min at 30 Hz. During the RNA extraction procedure the optional DNase treatment was carried out with the RNase-free DNase Set (QIAGEN, #79254) as described by the manufacturer. After extraction the concentration and purity of the RNA was determined by spectrophotometry (absorption at 260 nm and 280 nm). mRNA was reverse transcribed into cDNA using the ABI High Capacity cDNA Archive Kit (Applied Biosystems, #4322171). For total RNA extraction from mouse liver the RNeasy Mini Kit (QIAGEN, #74104) was used. Homogenization, DNase treatment and cDNA synthesis was done as described above. For RNA extraction from human brain/liver and cDNA synthesis the same procedures were used. Additionally two human RNAs from liver were ordered at Ambion (#7960) and Stratagene (#540017).

### Realtime-PCR gene expression analysis

Gene expression analysis was done by Realtime-PCR on an ABI PRISM 7700 Sequence Detection System (Applied Biosystems) [33]. TaqMan Gene Expression Assays were ordered from Applied Biosystems and Eurogentec or designed by us (see additional file 1: Assays used to quantify the expression of different complement components by Realtime-PCR in mice; and additional file 2: Assays used to quantify the expression of different complement components by Realtime-PCR in humans). The same cycle thresholds (Ct) were set for the different assays. Amplification was carried out with the qPCR MasterMix from Eurogentec (#RT-QP2X-03).

Quantification was done using the comparative Ct method (User Bulletin #2 ABI PRISM 7700 Sequence Detection System, Applied Biosystems), i.e. expression levels for the target genes were normalized to the 18srRNA of each sample [2^-ΔCt ^= 2^-(Ct(target gene)-(Ct(18srRNA))^]. Additionally two other housekeeping genes, HPRT and GAPDH, were analyzed to confirm the results as well as the integrity and quality of the cDNA (data not shown). Realtime-PCR quantifications were run in triplicate for each sample and the average determined. Mice were analyzed in groups of 8-12 per genotype. Human samples were analyzed likewise. Liver samples were used as positive control for all complement mRNA assays. In order to use the comparative Ct method for relative quantification the amplification efficiency of target and housekeeping gene must be approximately equal. All TaqMan Gene Expression Assays are optimized to 100% efficiency (+/- 10%) [34]. Self-designed primers and probes were analyzed for amplification efficiency using the Ct slope method. For this method a 6-log dilution range of target template was generated using 10-fold dilutions and the Ct value was determined. A plot of Ct versus log cDNA concentration was constructed. The optimal slope for this dilution series is -3.32 which results in a 100% amplification efficiency (E_x _= 1.0). A similar efficiency as for the TaqMan assays was obtained (data not shown).

### Western blot analysis

For Western blot analysis forebrains were homogenized in the Retsch Mill MM 300 followed by brief sonication to obtain a 1:10 dilution of forebrain homogenate in TBS complete (137 mM NaCl; 20 mM Tris/HCl pH 7.6; 1× complete (protease inhibitor cocktail tablet, Roche, #11836145001)). For gel electrophoresis an aliquot of forebrain homogenate or serum was diluted in SDS sample buffer. Proteins were separated by 8%, 12% and/or 15% SDS-PAGE (ratio of acrylamid/bisacrylamide 29:1) under reducing or non-reducing conditions (see additional file 3: Complement antibodies used on Western blots). They were transferred to polyvinylidene difluoride (PVDF) membranes (Millipore, #IPVH00010) by semi-dry blot. Membranes were blocked with 2% ECL Advance blocking agent (Amersham, #RPN2135) in PBST (Sigma, #P-3563). Antibodies were diluted in blocking solution and incubated for 1 h at room temperature or over night at 4°C. Detection of bound antibodies was carried out with peroxidase coupled secondary antibodies using the ECL Western blotting Detection system (Amersham, #RPN2106) or the ECL Advance Western blotting Detection System (Amersham, #RPN2135). For C1q and C3a detection the samples were analyzed under reducing conditions (with β-mercaptoethanol). For C3 analysis both conditions, reducing and non-reducing, were used. For quantification of C1q, different amounts of purified human protein (Calbiochem, #204876) were loaded on each gel along with the samples to be analyzed. The bands obtained on X-ray films were quantified using the program MCID Elite 7.0 (Imaging Research Inc.). Protein levels were read from standard curves generated with the Origin 7.5 software (OriginLab Corporation) by logistic curve fitting. The concentrations were expressed per weight of forebrain relative to the control group. A mouse C1q standard was not available and the potential difference in antibody affinity does not allow determination of absolute values. For quantification of C3 the purified human protein could not be used as a standard due to the species specificity of the antibody. An antibody recognizing both mouse and human C3 on Western blots was not available. Therefore the concentrations were expressed in arbitrary units.

### Immunoprecipitation (IP) of C3a from forebrain

For IP of C3a human brain samples and mouse forebrains were homogenized as described above to obtain 1:10 dilutions in TBS complete. To generate standard curves different amounts of purified human C3a protein (Cortex Biochem #CP1039) were added to non-transgenic forebrain homogenates for extraction and IP. Aliquots of 100 μl were used. After addition of NP40 (final concentration 0.1%) all samples were vortexed and incubated on ice for 15 minutes. During incubation they were mixed every 5 minutes. After centrifugation (15 minutes, 4°C, 20000 g) the cleared supernatant was transferred to a fresh tube and used for IP. IP was carried out using Dynabeads Protein G (Dynal, #100.03). For each sample 10 μl beads were used. Beads were resuspended by thoroughly vortexing and washed twice in 0.1 M Na-phosphate buffer, pH 5.8 using the Dynal MPC-S magnet (Dynal, #120.20). Finally the beads were resuspended in the original volume. For each 10 μl beads 5 μl of the C3a antibody was added to the prewashed beads, mixed and preincubated for at least 2 hours on a vertical end-over-end rotator. After preincubation the beads were washed again in 0.1 M Na-phosphate buffer pH 5.8 and then resuspended in the original volume. For IP 10 μl of washed antibody bound beads were added to the prepared samples and incubated over night at 4°C on a vertical end-over-end rotator. After incubation vials were placed on the magnet, the supernatant was removed and the beads were washed in 1% NP40 in TBS complete followed by 10 mM Tris (pH 7.5) and 1 mM Tris (pH 7.5). After each washing step the supernatant was removed on the magnet. Following the last washing step the samples were briefly spun down and the remaining supernatant was collected on the magnet. Finally the beads were resuspended in 10 μl 1.3× Laemmli sample buffer for Western blot analysis.

### Histology

Immersion fixed brains from APP23 mice at 20 months of age and pieces from the frontal pole (F2) of two AD brains (1^st^: male, aged 74, CERAD C, Braak IV; 2^nd^: female, aged 82, CERAD C, Braak V) were embedded into paraffin and cut with a microtome into sections of 4 μm. Sections were deparaffinized in xylene and rehydrated in a descending concentration of ethanol in H_2_O. Antigenity was enhanced by microwave heating of sections in 0.1 M citric acid buffer at 90°C for 60 minutes. Tyramide Signal Amplification (TSA plus) immuno fluorescence histochemistry was performed using a commercial kit (TSA-direct kit, NEN Life Science, Perkin Elmer). Different antibodies were tested to localize complement components in brain (see additional file 4: Complement antibodies used in histology). Sections were incubated for 24 h at 4°C with primary antisera/antibodies, diluted in TNT (0.3% Triton X-100, 100 mM Tris, 150 mM NaCl). Negative controls included omission of the primary antisera and substitution by different non-immune sera. After incubation the sections were rinsed in TNT (6 × 5 min) and incubated at RT for 1 h in secondary antibody solution. The horseradish peroxidase (HPR, DAKO) labelled secondary antibody was diluted 1:200 in TNB (0.5% blocking solution TSA Kit NEL 741). After 6 × 5 min TNT washing sections were incubated for 15 min in FITC-Tyramide solution (1:200 dilution in amplification buffer according manufactures conditions). After washing in TNT and transferring to Super Frost Plus glass slides (Menzel, Heidelberg) sections were embedded in Vectashield (Vector, H-1000) for Fluorescence microscopy. Sections were examined and photographed using fluorescence microscopy with the following filter combination: excitation 450-490 nm, emission 510 nm.

### Statistical analysis

The averages were calculated for all groups as the data were normally distributed for most of the groups. Calculation of medians gave very similar results. At each age the transgenic group was compared to the corresponding wildtype control group, i.e. pair wise comparison to the control group was done using 2-tailed Student's t-test (p < 0.05 considered significant). In the kinetic analyses we compared the transgenic and non-transgenic groups at each age using 2-tailed Student's t-test. In addition for both genotypes each age was compared to the corresponding 3 months group using Dunnetts test.

## Results

### mRNA expression of complement components in amyloid plaque-containing APP23 and control mouse brain

APP23 mice show an age-related increase in amyloid plaque deposition as well as in plaque-associated alterations such as gliosis. We, therefore, initially compared 24 months old female APP23 with age-matched non-transgenic mice to identify alterations in expression of key complement components in forebrain by Realtime-PCR. The values given for all complement mRNAs are normalized to 18srRNA; very similar results were obtained by normalization to the housekeeping genes HPRT or GAPDH.

The mRNAs of only some complement components were substantially and significantly elevated relative to non-transgenic mice. These include the initiator of the classical pathway C1q, the central component C3, which also initiates the alternative pathway, and an alternative pathway inhibitor, factor H. The increase of factor B, a specific member of the alternative pathway, just did not reach significance (Table 1). Other components, C4, C5 and the inhibitor Crry, were unchanged. Only components of the early complement activation steps were increased.

**Table 1:** Brain mRNA expression of complement components in APP23 and non-transgenic controls at 24 months

	pathway/function	control mice ± SEM	APP23 ± SEM	p (2 tailed t-test)	increase APP23
**C1q**	classical pathway	89'040 ± 4'788	189'896 ± 7'721	0.000000006	2×

**C4**	classical pathway	10'042 ± 1'104	11'016 ± 1'754	0.64	1×

**factor B**	alternative pathway	647 ± 92	1'029 ± 164	0.06	1.6×

**C3**	all pathways	3'317 ± 114	10'550 ± 1'507	0.0007	3

**C5**	terminal sequence	117 ± 11	128 ± 12	0.51	1×

**C6**	terminal sequence	13 (below limit of quantification)	43 (below limit of quantification)	-	-

**C8a**	terminal sequence	not detectable	not detectable	-	-

**C8b**	terminal sequence	32 (below limit of quantification)	46 (below limit of quantification)	-	-

**C9**	terminal sequence	not detectable	not detectable	-	-

**Crry**	inhibitor all pathways	48'546 ± 4'156	49'891 ± 3'120	0.80	1×

**MCP**	inhibitor all pathways	20 (below limit of quantification)	37 (below limit of quantification)	-	-

**factor H**	inhibitor alternative pathway	14'756 ± 723	21'509 ± 913	0.00001	1.5×

Values are given as arbitrary units.

The levels of the complement mRNAs seemed to differ considerably for the different components (Table 1). Although a direct comparison of the units determined is not possible, all assays were shown to have similar efficiencies (see methods) and it appears unlikely that systematic differences of about an order of magnitude are due to assay differences. The highest levels were found for both components of the classical complement pathway, C1q and C4, while far lower values were determined for factor B, representing the alternative pathway. C3 showed a relatively strong expression, but the precursor of the second convertase C5 was much less expressed. The RNAs of terminal sequence components C6 and C8b could be measured but were below the limit of quantification. C8a and C9 mRNA remained below the limit of detection in control and APP23 mice. The inhibitory complement components factor H and Crry were remarkably well expressed. In comparison, MCP RNA was low, which may be due to the fact that in mice the function of MCP is mainly carried out by Crry [35,36].

We also analyzed a group of perfused 25 months old APP23 mice to test if the blood remaining in brain might influence the results. Very similar expression levels were found for C1q, C3, C4, C5 and factor B mRNA (data not shown).

### Age-dependent complement expression in non-transgenic mouse brain

To evaluate changes in complement expression with normal aging we determined the mRNA levels of all studied components in young non-transgenic mice (3 months of age). Comparison with the expression levels in 24 months old mice showed a substantial increase with age for C3 and C4, while C1q, factor B and C5 were elevated to a smaller degree (Table 2). The later components of the terminal sequence showed very low if any expression in young as well as old animals. The inhibitory factors tested also did not seem to change much with age. We also analyzed APP23 mice at 3 months of age and found no difference in expression compared to non-transgenic animals (data not shown). An exception was C4 as its expression was lower in young APP23 than in non-transgenic mice (see below).

**Table 2:** Age dependent increase of complement expression in non-transgenic mouse brain

	3 months	SEM	24 months	SEM	p (2 tailed t-test)	increase with age
**C1q**	28'345	2'339	89'040	4'788	0.0000001	3×

**C4**	1'113	155	10'042	1'104	0.00003	9×

**factor B**	224	28	647	92	0.0009	3×

**C3**	349	38	3'317	114	0.0000000004	9×

**C5**	68	7	117	11	0.00001	2×

**C6**	6	-	13	-	-	below limit of quantification

**C8a**	not detectable	-	not detectable	-	-	-

**C8b**	41	-	32	-	-	below limit of quantification

**C9**	not detectable	-	not detectable	-	-	-

**Crry**	39'007	1'672	48'546	4'156	0.05	1×

**MCP**	15	3	20	2	-	below limit of quantification

**factor H**	16'861	1'532	14'756	723	0.24	1×

Values are given as arbitrary units.

Very low complement activity has been claimed for standard laboratory mouse strains including C57BL/6, the background strain of APP23, while much higher activity was found in BUB/BnJ mice [32,37]. To test if this strain might provide a background more favourable for complement activation we determined the expression of C1q, C3, C5, C6 and C9 in forebrain of male BUB/BnJ mice but found no major differences compared to C57BL/6 (data not shown).

### Complement mRNA expression in mouse liver

Liver is the main source of the complement components in the body and we determined the expression of the most important factors in order to compare with brain (see additional file 5: Expression of complement components in the liver of APP23 mice). All components were expressed well in liver including C9, which was easily detected. Except for C1q, which showed the same expression as in brain, all other components reached much higher mRNA levels in the liver. Consequently, the ratio between the different complement mRNAs was very different in brain and liver.

### Complement mRNA expression in human brain and liver

Complement factors up to C9 have been found in human brain [2-4]. We analyzed cerebral cortex from 3 aged but non-demented donors for complement expression (see additional file 6: Complement mRNA expression in human cerebral cortex and liver). C1q mRNA levels were in the range of those found in mice. In contrast, C3 and C5 showed considerably higher levels in human brain. Similar to mouse brain, C6 expression was low and C9 expression was not detectable in human brain. The mRNAs of the inhibitory components factor H and MCP were in the range of mouse brain considering that Crry and MCP are functional homologs. Similar to mouse, human liver showed high expression of all tested complement components.

### Brain C1q, C3, C4 and factor H and B mRNA expression during aging and amyloid deposition

The main complement components with altered brain expression in old APP23 mice were C1q, C3, factor H and possibly factor B, whereas C4 seemed reduced in young transgenic animals. The expression kinetics of these components were analyzed in forebrain of female APP23 and control mice at 3, 6, 9, 12, 15, 18, 24 and 30 months using Realtime-PCR.

**Figure 1:**
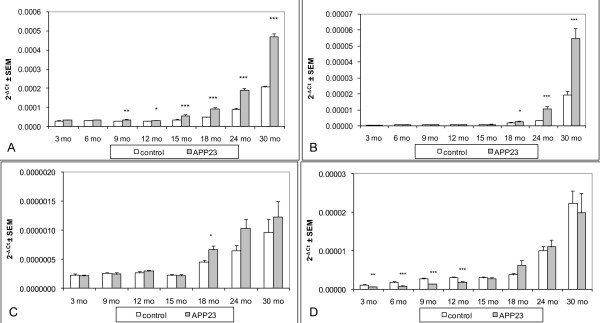
**Brain C1q, C3, factor B, C4 expression during aging in APP23 and control mice**. Pair-wise comparison of transgenic and non-transgenic groups at each age using the 2-tailed t-test (* p = 0.05-0.01; ** p = 0.01-0.001; *** p = < 0.001); **A**: C1q expression (comparison of 3 months group with aged groups using Dunnetts test showed significance (p < 0.05) from 15 months (APP23) and 18 months (controls) onwards); **B**: C3 expression (comparison of 3 months group with aged groups showed significance from 24 months onwards (APP23 and controls)); **C**: factor B expression (comparison of 3 months group with aged groups showed significance from 18 months (APP23) and 24 months (controls) onwards); **D**: C4 expression (comparison of 3 months group with aged groups showed significance from 24 months onwards (APP23 and controls)).

C1q expression remained similar in APP23 and non-transgenic mice at 3 and 6 months (Figure 1A). An increase over age-matched controls was detectable at 9 months when the first amyloid deposits had formed. This increase became larger during further aging reaching a factor of ~2, which was not exceeded much even at 30 months of age. However, compared to baseline at 3 months C1q expression in APP23 mice had increased 14-fold at 30 months. Non-transgenic mice also showed a considerable elevation of C1q expression during aging (7-fold at 30 months). The age-related elevation in non-transgenics explains the relatively small increase when APP23 mice are compared to age-matched controls.

During aging C3 increased in non-transgenic mice (~56-fold at 30 months compared to 3 months) and much stronger in APP23 mice (~140-fold) (Figure 1B). No difference between APP23 and non-transgenic mice was detectable for C3 until 15 months of age. Compared to age-matched controls an elevation of ~3-fold was reached at 24 and remained at 30 months of age. This elevation started much later than for C1q. It matched in time with the small increase over age-matched controls of factors B and H, a component and an inhibitor, respectively, of C3 convertase in the alternative pathway. For factor B the elevation over non-transgenic mice started at 18 months (1.5-fold) but was small (24 months: 1.6-fold, 30 months: 1.3-fold) (Figure 1C). Compared to 3 months factor B expression also increased during aging in both APP23 and control mice. Factor H expression as well was first elevated in APP23 mice compared to age matched controls at 18 months reaching an about 1.5-fold increase at 24 months of age (data not shown). Non-transgenic mice did not show a consistent age-related expression change.

A brain expression increase with age was also found for C4 in APP23 (~34-fold) and control (~20-fold) mice (significant from 24 months on, (Figure 1D)). However, APP23 started at a significantly lower expression level (0.5-fold) than non-transgenic controls. This difference was lost at 15 months indicating a transgene-induced elevation of expression, which adds to the age-related increase in non-transgenic mice. The reason for the lower C4 mRNA expression in young APP23 transgenic mice remains unclear.

### Complement C1q and C3 protein in brain of aging APP23 and control mice

We next attempted to detect the complement proteins in mouse brain homogenates to confirm the increases found on the RNA level. Among several antibodies tested, suited ones reacting with mouse complement components on Western blots were only found for C1q, C3 and C3a.

**Figure 2:**
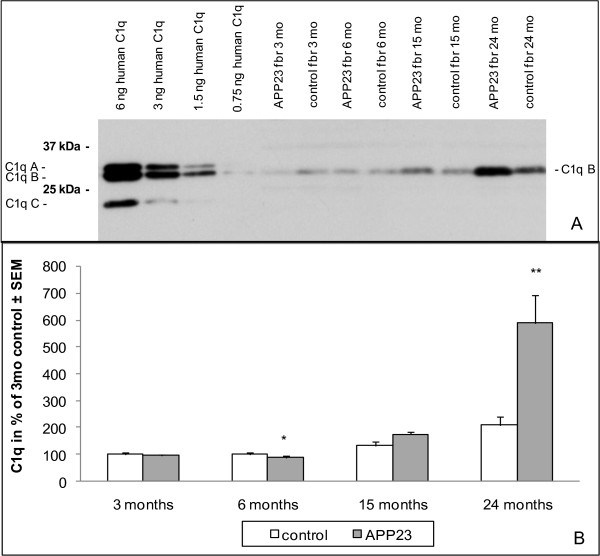
**Age dependency of C1q protein levels in APP23 and control mouse forebrain**. **A**: Representative Western blot of APP23 and control forebrain at different ages as well as purified human C1q protein standards. Proteins were separated by 12% SDS-PAGE under reducing conditions, detected with a goat C1q antibody (Calbiochem): **B**: Quantification of C1q from Western blot analysis of APP23 and control forebrain at different ages (n = 8). Transgenic and non-transgenic groups were compared at each age using the 2-tailed t-test (* p = 0.05-0.01; ** p = 0.01-0.001; *** p = < 0.001); comparison of the 3 months groups (APP23 and controls) with aged groups using Dunnetts test showed significant increases (p < 0.05) at 24 months.

In mouse brain the C1q antibody specifically detected one band at 26 kDa corresponding to the C1q B chain (Figure 2A). It did not react well with chain A and C. In control mice (C57BL/6) the C1q band increased with age. Aged APP23 showed an additional elevation over age-matched controls. There was no difference in the intensity of the C1q signals between PBS perfused and non-perfused forebrains indicating little contribution from blood. To quantify the increase in C1q expression on the protein level, groups of APP23 (n = 8) and control forebrains (n = 8) at 3, 6, 15 and 24 months were analyzed by Western blotting. The C1q B chain at 26 kDa was quantified using the corresponding band from purified human C1q as standard (Figure 2B, see also methods). Brain C1q in C57BL/6 mice remained constant between 3 and 6 months but showed an age-dependent increase starting at 15 months reaching significance at 24 months. At 3 months of age no difference in the C1q forebrain concentration could be detected between APP23 and control mice. There was a small just significant reduction of C1q in the APP23 group at 6 months of age which failed to reach significance in a second quantification. At 15 months APP23 mice showed a non-significant C1q elevation of 31%, which rose to a significant increase of 181% at 24 months. These results are in good agreement with the data found on the mRNA level.

**Figure 3:**
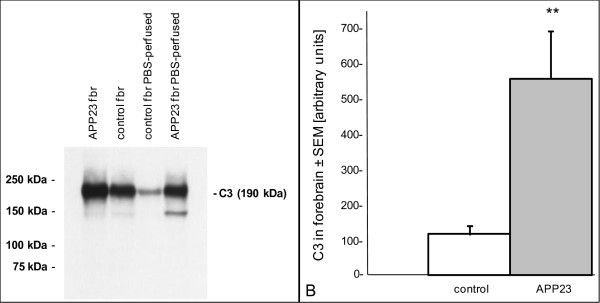
**C3 protein in perfused and non-perfused APP23 and control forebrain**. **A**: Western blot analysis of APP23 and control forebrain at 24 months of age (PBS-perfused and non-perfused). Proteins were separated by 8% SDS-PAGE under non-reducing conditions and detected with a goat anti-C3 antibody (Cappel); **B**: Quantification of C3 in PBS-perfused forebrains of APP23 and control mice at 24 months of age.

C3 could be detected in forebrain, serum and liver of control mice and was notably increased in brain of 24 months old APP23 compared to age matched controls. After PBS perfusion of the mice the intensity of the C3 band decreased both in APP23 and control mice which indicated that part of the C3 signal in non-perfused forebrains originated from the remaining blood (Figure 3A). In contrast to C1q, where residual blood could be neglected (see above), C3 reaches a lower concentration in brain but a higher one in plasma resulting in detectable contamination of non-perfused brain samples. To estimate the change in C3, groups (n = 9) of PBS-perfused APP23 and non-transgenic forebrains at 25 months of age were analyzed by Western blotting. In APP23 mice C3 was increased almost 4 fold compared to controls (Figure 3B). This was in good agreement with the mRNA quantification results.

The major complement amplification step includes C3 cleavage generating C3a and C3b. To obtain evidence for this activation we attempted to immunoprecipitate C3a followed by Western blot detection. Purified human C3a protein was recognized by a rabbit anti-human C3a antibody at 9 kDa. This antibody also reacted with a distinctive band in human brain and serum as well as in APP23 and control serum. In perfused APP23 forebrain, however, no specific band could be detected.

### Immunohistochemical localization of complement components in APP23 and AD brain

**Figure 4:**
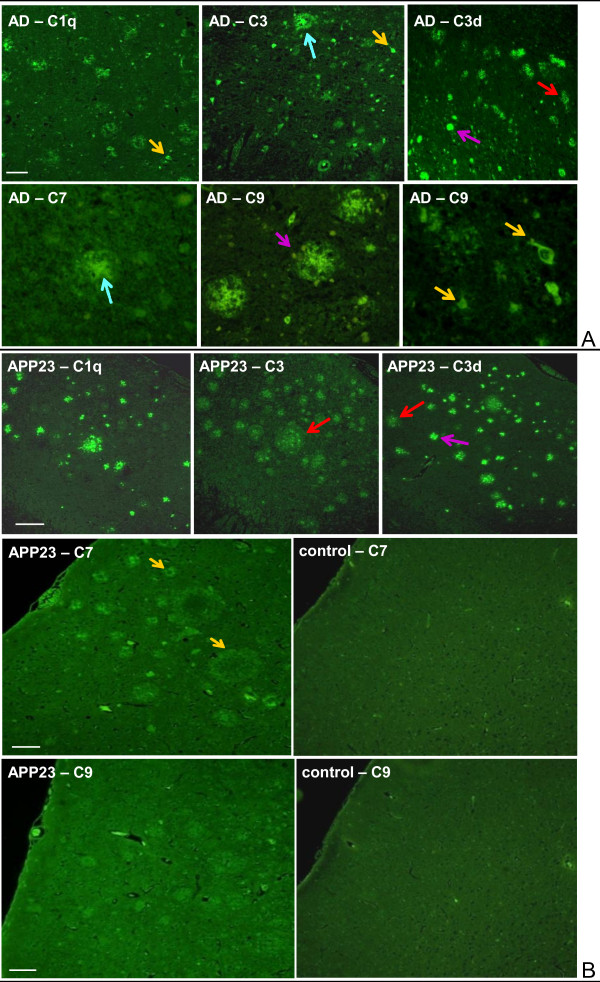
**Complement immunostaining of AD and APP23 mouse brain**. **A**: Neocortex (F2) of an AD brain: C1q, C3, C3d, C7 and C9 immunostaining of diffuse (red arrow), compact (violet arrow) and cored (blue arrow) amyloid plaques and neurofibrillary tangles (yellow arrow); bar = 50 μm; **B**: Neocortex of a 20 months old female APP23 mouse: C1q, C3 and C3d immunostaining of diffuse (red arrow) and compact (violet arrow) amyloid plaques; C7 immunostaining of the amyloid plaque periphery (yellow arrow); C9 staining: weak fluorescence above background (control), no specific staining of non-transgenic mice (control); bar = 100 μm.

Using the highly sensitive TSA detection method immunohistochemical staining of Alzheimer brain tissue was obtained with antibodies to proteins associated with the classical complement pathway (Clq, C3, C3d) and the terminal sequence (C7 and C9) (Figure 4A). These antibodies stained senile plaques, dystrophic neurites and some neurofibrillary tangles as previously described [21]. The C7 antibody showed rather weak staining of some amyloid plaque cores only. In contrast, C9 labelling of plaques and tangles was relatively strong, which has been taken as evidence of a full activation of the complement cascade in human brain [13,21].

In APP23 mice complement proteins C1q, C3 and C3d were found strongly associated with compact amyloid deposits (Figure 4B). C3d staining demonstrated some activation of the key amplification step. As compared to humans the recruitment of early complement components was similar. Unlike human AD brain C7 was absent from the amyloid plaque core but could be detected as weak staining in the plaque periphery, which likely reflects an association with cellular elements. C9 remained very weak or negative. Although the antibody recognized murine C9 on Western blots of serum a reduced reactivity on mouse brain as compared to human histological sections cannot be excluded. Non-transgenic mice did not show any specific staining.

## Discussion

Considerable histological evidence has been obtained indicating a full activation of the complement cascade up to the membrane attack complex in human AD brain. In contrast, decoration of amyloid plaques in APP transgenic mouse models has been limited to early components. To better understand the contribution of age and amyloid deposition, we have analyzed the expression of complement components during aging and their induction by amyloid deposition in brain of wildtype and plaque-forming APP transgenic mice.

Expression of early complement components was detectable already in young C57BL/6 wildtype animals. During aging, in particular after 15 months, the levels increased considerably but disproportionate thus changing the ratio between the different mRNAs (C5: 2×, C1q and factor B: 3×, C3 and C4: 9× between 3 and 24 months). Our data demonstrate an age-related increase in RNA expression for both, the classical and alternative complement pathways. In pre-plaque APP23 mice, complement expression did not differ from the C57BL/6 controls an exception being a reduction in C4. At 24 months some of the complement RNAs were elevated beyond the aged control mice (factor B: 1.5×, C1q: 2×, C3: 3×), while C4 now reached wildtype levels. The transgene-related increases were generally smaller than the age-related elevations. These data are consistent with the increased C3 expression described in the J20 APP transgenic mouse line [27]. Interestingly, in a mouse model for cerebral microvascular Aβ deposition C1q, C3 and C4 have also been found elevated [38]. The kinetics of complement RNA induction were very similar for APP23 and C57BL/6 mice. The earliest mRNA increase was found for C1q at 9 months of age. A significant elevation of C4 mRNA could only be detected at 15 months followed by C3 and factor B RNAs at 18 months. Quantification of C1q and C3 polypeptides demonstrated similar increases thus confirming the changes of the major factors on the protein level. These data show that the elevation of early complement expression in mouse brain is primarily age-related with only a smaller additional response to amyloid deposition. The known increase and activation of glia during amyloid deposition in APP23 mice [39] may contribute to this further elevation. Interestingly, neither the age- nor the amyloid-related increase reached a plateau as may be expected. This could be related to the lack of full complement activation in conjunction with missing negative feedback.

In contrast to the early complement components, brain mRNA levels of the final ones following C5 were very low or not detectable and remained unchanged during aging of both, C57BL/6 and APP23 mice. Among all activating complement components analyzed, only C1q expression reached levels comparable to liver, whereas the others remained considerably lower. The BUB/BnJ mouse strain, claimed to show higher complement activity than the C57BL/6 strain used here [32,37], did not differ in brain complement expression. This is in line with the recent demonstration of a similar serum complement activity as other mouse strains [40]. This strain, therefore, does not seem suited to promote brain expression of the final complement components.

Our preliminary analysis of a small number of aged human brain samples also indicates a reduced expression towards the later complement components including a lack of detectable C9 mRNA. However, the human brain expression levels in particular of C3 and C5 were considerably higher than mouse brain expression. Previous studies moreover indicated a substantial mRNA up-regulation for all classical and the alternative pathway component factor B in AD brain [16,41]. This increase in expression of components required to activate complement is in clear contrast to our observations in APP transgenic mice, where complement mRNA upregulation is restricted to early components.

Interestingly, high mouse brain expression was found for the inhibitors Crry and factor H, which block formation of the C3 convertase. The levels were similar in wildtype and APP23 transgenic mice, remained stable throughout aging and were comparable to human brain considering MPC as the functional Crry analog. Irrespective of these high inhibitor levels some C3 convertase activation still seems to occur. Otherwise overexpression of soluble Crry and deletion of C3, which increased Aβ deposition and neurodegeneration [26,27], should not have led to any changes. Similarly, the beneficial effect of the C5aR antagonist on the APP transgenic mouse pathology [30] can only be observed in the presence of some C5 activation. However, interference at C3 and C5 in humans may have a different outcome than in mice owing to the different expression levels and activator/inhibitor ratios.

Using AD brain sections we could easily detect C1q, C3, C3d, C7 and C9 at amyloid plaques and neurofibrillary tangles confirming previous data [10,13-15,20,21]. Similar staining of the early complement components C1q, C3 and C3d was found in aged APP23 mouse brain. This is at variance from Schwab and co-workers [21] who described a general decrease in complement protein staining in APP23 mice. The difference may be explained by a lower reactivity of their antibodies with the mouse proteins. The generation of C3d indicates some activation of C3 convertase. Nonetheless, the downstream component C7 was not associated with the amyloid plaque center but weakly detected in the periphery, likely in cellular elements. Consequently, C9 staining could not be found in APP23 mice. This lack of plaque decoration by the downstream complement components contrasts the human situation and argues against a comparable activation.

## Conclusion

Overall, our data indicate a low complement expression in mice from the C3 component onwards. Starting at C5 the mRNAs did not increase in response to amyloid deposition, which is in contrast to human brain. While we have not detected a clear immunohistological difference to human AD brain at the C3 level, the reduced expression matches with the lack of deposition of the downstream components on amyloid plaques in mice. The low expression may hence contribute to the restricted complement activation but it is not restricted to the loss of a specific component.

## Abbreviations

Aβ: β-amyloid; AD: Alzheimer's disease; APP: Amyloid precursor protein; Crry: Complement receptor 1-related protein y; Ct: Cycle threshold; fbr: forebrain; GAPDH: Glyceraldehyde phosphate dehydrogenase; HPRT: Hypoxantine phosphoribosyltransferase; IP: immunoprecipitation; MAC: membrane attack complex; MCP: Membrane cofactor protein; TSA: Tyramide Signal Amplification.

## Competing interests

The authors declare that they have no competing interests.

## Authors' contributions

JR carried out most of the experimental and statistical work and helped to draft the manuscript. SD carried out the histological analyses. KHW was responsible for the immunohistological work. MS conceived and designed the study and drafted the manuscript. All co-authors contributed to the preparation of the manuscript. All authors read and approved the final manuscript.

## Supplementary Material

Additional file 1 **Assays used to quantify the expression of different complement components by Realtime-PCR in mice**. Tabular data of the Realtime-PCR assays used to quantify the complement expression in mice. Click here for file

Additional file 2 **Assays used to quantify the expression of different complement components by Realtime-PCR in humans**. Tabular data of the Realtime-PCR assays used to quantify the complement expression in humans. Click here for file

Additional file 3 **Complement antibodies used on Western blots**. Tabular data of the antibodies used for Western blot analyses including dilutions and ordering information. Click here for file

Additional file 4 **Complement antibodies used in histology**. Tabular data of the antibodies used for histological analyses including dilutions and ordering information. Click here for file

Additional file 5 **Expression of complement components in the liver of APP23 mice**. Tabular data of the complement expression in liver of APP23 mice. Click here for file

Additional file 6 **Complement mRNA expression in human cerebral cortex and liver**. Tabular data of the complement expression in human cerebral cortex and liver. Click here for file
